# High-accuracy wavefront sensing by phase diversity technique with bisymmetric defocuses diversity phase

**DOI:** 10.1038/s41598-017-15597-x

**Published:** 2017-11-10

**Authors:** Peiguang Zhang, Chengliang Yang, Zihao Xu, Zhaoliang Cao, Quanquan Mu, Li Xuan

**Affiliations:** 1 State Key Laboratory of Applied Optics, Changchun Institute of Optics, Fine Mechanics and Physics (CIOMP), Chinese Academy of Sciences, Changchun, Jilin, 130033 China; 2 Graduate School of the Chinese Academy of Science, Beijing, 100039 China

## Abstract

We investigate a specific diversity phase for phase diversity (PD) phase retrieval, which possesses higher accuracy than common PD, especially for large-scale and high-frequency wavefront sensing. The commonly used PD algorithm employs the image intensities of the focused plane and one defocused plane to build the error metric. Unlike the commonly used PD, we explore a bisymmetric defocuses diversity phase, which employs the image intensities of two symmetrical defocused planes to build the error metric. This kind of diversity phase, named PD-BD (bisymmetric defocuses phase diversity), is analysed with the Cramer-Rao lower bound (CRLB). Statistically, PD-BD shows smaller CRLBs than the commonly used PD, which indicates stronger capacity of phase retrieval. Numerical simulations also verify that PD-BD has higher accuracy of phase retrieval than the commonly used PD when dealing with large-scale and high-frequency wavefront aberrations. To further affirm that PD-BD possesses higher accuracy of wavefront sensing than PD, we also perform a simple verification experiment.

## Introduction

Phase diversity (PD) algorithm was firstly introduced by Gonsalves and Chidlaw^1^, which is a well-known algorithm of phase retrieval (PR) algorithms. This algorithm requires the image intensities of a simultaneous set of focused image and defocused image to build the error metric used in numerical optimization processing. The focused image is the target image degraded by system aberration, while the defocused image is the same blurred image, but with an additional known defocus aberration. The reconstruction of unknown wavefront aberration can be obtained by minimizing the error metric. PD algorithm could achieve near-diffraction-limited imaging and it is suitable for point target and extended target at the same time. PD algorithm is mainly used in high resolution imaging^2,3^, and adaptive optics (AO) system^4–10^, Other applications of PD algorithm also include measurement of laser beams^11,12^, fresnel incoherent holography^13^ and biological microscopy imaging^14^.

The accuracy of phase reconstruction is closely linked with the optimization algorithm selected and the characteristics of PD physical model. Gradient-based optimization algorithms used in PD, such as conjugate-gradient algorithm(CG)^15,16^, and Broyden-Fletcher-Goldfarb-Shanno (BFGS) algorithm^17,18^, are easily trapped in local minimums near the initial positions. Based on a reliable global optimization algorithm, such as hybrid particle swarm global optimization algorithm^19^, the accuracy of PD is then determined by the behavior of PD physical model. Scholars have made some adjustments on the physical model of traditional PD to improve its accuracy, such as multiframe phase-diversity joint estimation^20^ and phase diversity speckle (PDS) algorithm^17^. The commonly used diversity phase of traditional PD is a known defocus phase. A mixed diversity phase composed of astigmatism and focus has been empirically selected to improve the accuracy of PD^21^. Phase diversity technique has also been introduced to blind deconvolution image restoration^22.23^.

The diversity phase is a phase relative to the measured system aberration, which provides a helpful constraint condition for the reconstruction of the measured system aberration. The commonly used diversity phase of PD employs the image intensities of the focused plane and one defocused plane to build the error metric. The measured system aberration within the focused plane can be seen as a zero-diversity phase. And the defocused plane contains a known defocus diversity phase relative to the measured system aberration. In this paper, we investigate a specific diversity phase for PD phase retrieval. The main idea is to remove the zero-diversity phase and to add a symmetric defocus diversity phase. The bisymmetric defocuses diversity phase, named PD-BD (bisymmetric defocuses phase diversity), employs the image intensities of two symmetrical defocused planes to build the error metric. Obviously, compared with traditional PD, PD-BD introduces no additonal computational burden. PD-BD was first introduced by Lee *et al*. (1999)^24^, which was analyzed by Cramer-Rao lower bound. Their work hinted that PD-BD might possess a better performance for wavefront sensing. In general, PD-BD could be seen as a special case of the multi-image PD algorithm presented by Paxman *et al*. (1992)^20^, derived for an arbitrary number of diversity images. In our opinion, two bisymmetric defocus diversity phases can provide helpful constraint conditions for the reconstruction of the measured system aberration.

## Results

### CRLB analysis and numerical simulations

PD is a posteriori image-based aberration estimation technique. The smallest possible unbiased estimator variance for PD technique can be theoretically given by the Cramer-Rao lower bound (CRLB). CRLB could provide a lower bound on the mean-square error of an estimator, which can be used to theoretically evaluate the accuracy of wavefront sensing by phase diversity technique. The fundamental concept of CRLB is written symbolically as

1 $$Var({\tilde{a}}_{k})\ge {[{{\bf{F}}}^{-1}({\boldsymbol{a}})]}_{kk},$$

where ${\tilde{a}}_{k}$ denotes an unbiased estimate of *a*_*k*_. The *Var*(·) operator denotes the variance of the ${\tilde{a}}_{k}$. The symbol [*F*(***a*** **)**] stands for the Fisher information matrix of ***a***. We use the same definition of CRLB as ref.^25^, of which the CRLB is denoted as the corresponding square root that bounds the rms error of an estimator. So the CRLB of PD technique on a Zernike parameter *a*_*k*_ is expressed as

2 $$CRLB({a}_{k})=\sqrt{{({{\bf{F}}}^{-1})}_{kk}}\mathrm{.}$$

The equality states that the estimated parameter vector ***a*** is bounded from below by the corresponding square root of diagonal elements of a Fisher information matrix inverse. Detailed expression of the Fisher information matrix ***F*** and descriptions for the CRLB of PD can be obtained in Refs^24,25^. We suppose a high signal-to-noise ratio (SNR) for the computation of CRLB. In this paper, we will consider the CRLBs of PD and PD-BD for the simulated object under the assumption of Gaussian noise.

**Figure 1:**
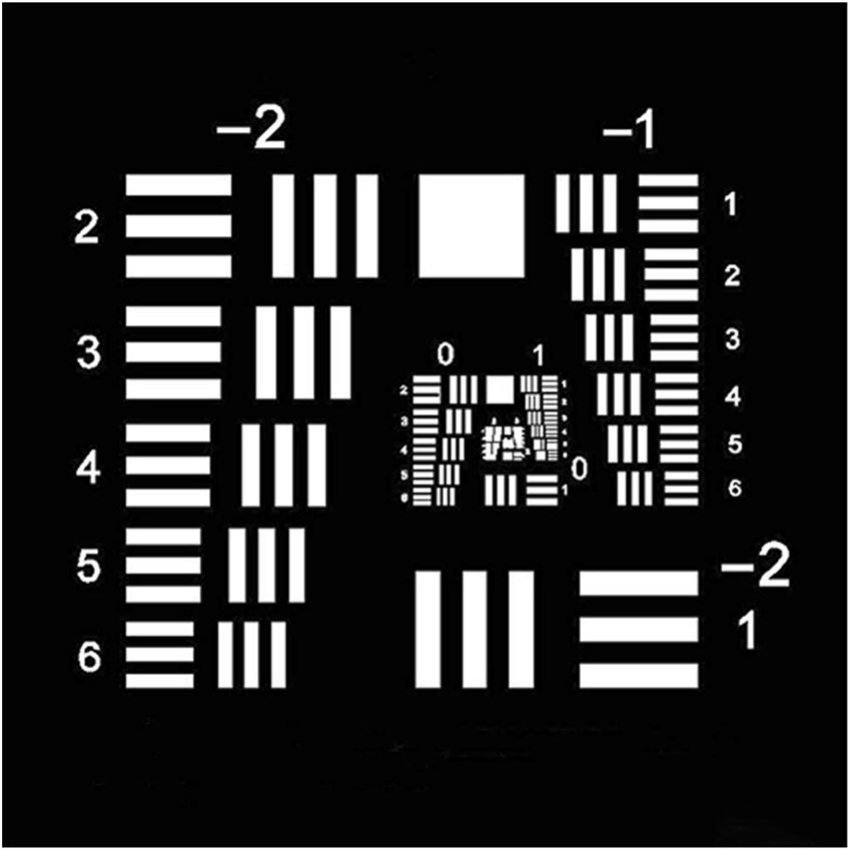
The pristine object used for CRLB analysis and numerical simulations.

We will carry on the comparisons between PD and PD-BD by CRLB analysis and numerical simulations. The pristine object used for CRLB analysis and numerical simulations is shown in Fig. 1. The object here is a high signal-to-noise ratio (SNR) object which will help to approach theoretical CRLB. In fact, it is unimportant to know accurate SNR when we focus on the comparison of CRLB between PD and PD-BD. The size of the Nyquist sampled object is 200 × 200 pixels and the intensity levels are quantized to 16 bits. The size of simulated pupil is 100 × 100 pixels. For numerical simulations, the regularization parameter *γ* is set to be 1.0 × 10^−6^. The optimization algorithm used here is a hybrid particle swarm optimization algorithm^19^, which can provide global search and avoid being trapped in local minimums. The amount of defocus used for PD is commonly set to be 0.5*λ* PV or 1.0*λ* PV (peak to valley). In the following, we will show that the amount of symmetrical defocuses used for PD-BD can be set as ±0.5*λ* PV, ±0.75*λ* PV and ±1.0*λ* PV, which possess similar performance. We denote PD with 0.5*λ* PV and 1.0*λ* PV defocus as PD-0.5 and PD-1.0, respectively. Similarly, PD-BD with ±0.5*λ* PV, ±0.75*λ* PV and ±1.0*λ* PV defocus are denoted as PD-0.5, PD-0.75 and PD-1.0, respectively. The measured wavefront aberration is denoted as *ϕ*_*set*_. The rms value of *ϕ*_*set*_ is defined as follows:

3 $$RMS(\lambda )=\frac{1}{2\pi }{[\sum _{v}{({\varphi }_{set}({\bf{v}})-{\varphi }_{aver}({\bf{v}}))}^{2}/{N}_{T}]}^{\frac{1}{2}},$$

where *v* is the two-dimensional position coordinate in the pupil plane. *ϕ*_*aver*_ is the average value of *ϕ*_*set*_, *N*_*T*_ is the total number of pixels within the pupil aperture. The accuracy of the reconstructed wavefront aberration *ϕ* is quantified with the rms phase error:

4 $$RMS\,\,{\rm{error}}(\lambda )=\frac{1}{2\pi }{[\sum _{v}{(\varphi ({\bf{v}})-{\varphi }_{set}({\bf{v}}))}^{2}/{N}_{T}]}^{\frac{1}{2}}\mathrm{.}$$

**Figure 2:**
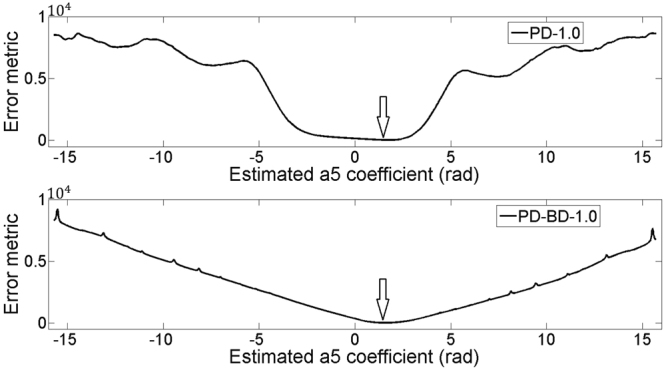
Error metrics plotting of PD-1.0 and PD-BD-1.0 with a astigmatism system aberration *a*_5_ = 1.5 radian.

We first compare the error metrics of PD-1.0 and PD-BD-1.0 with a astigmatism system aberration *a*_5_ = 1.5 radian. The error metrics of PD-1.0 and PD-BD-1.0 then reduce to functions of one single variable *a*_5_, which are explicitly plotted in Fig. 2. We can see that PD-BD-1.0 provides a steeper gradient than PD-1.0 near the minimum extreme point *a*_5_ = 1.5. Steeper gradients near the global minimum position mean a easier search for the measured phase with a higher accuracy.

**Figure 3:**
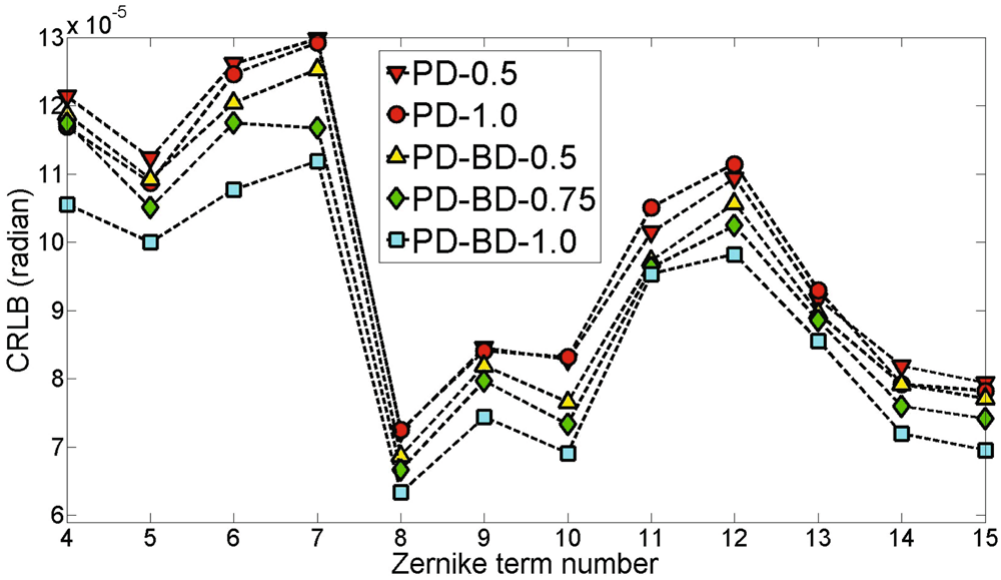
CRLBs plotting of PD and PD-BD on the aberration listed in Table 1.

**Figure 4:**
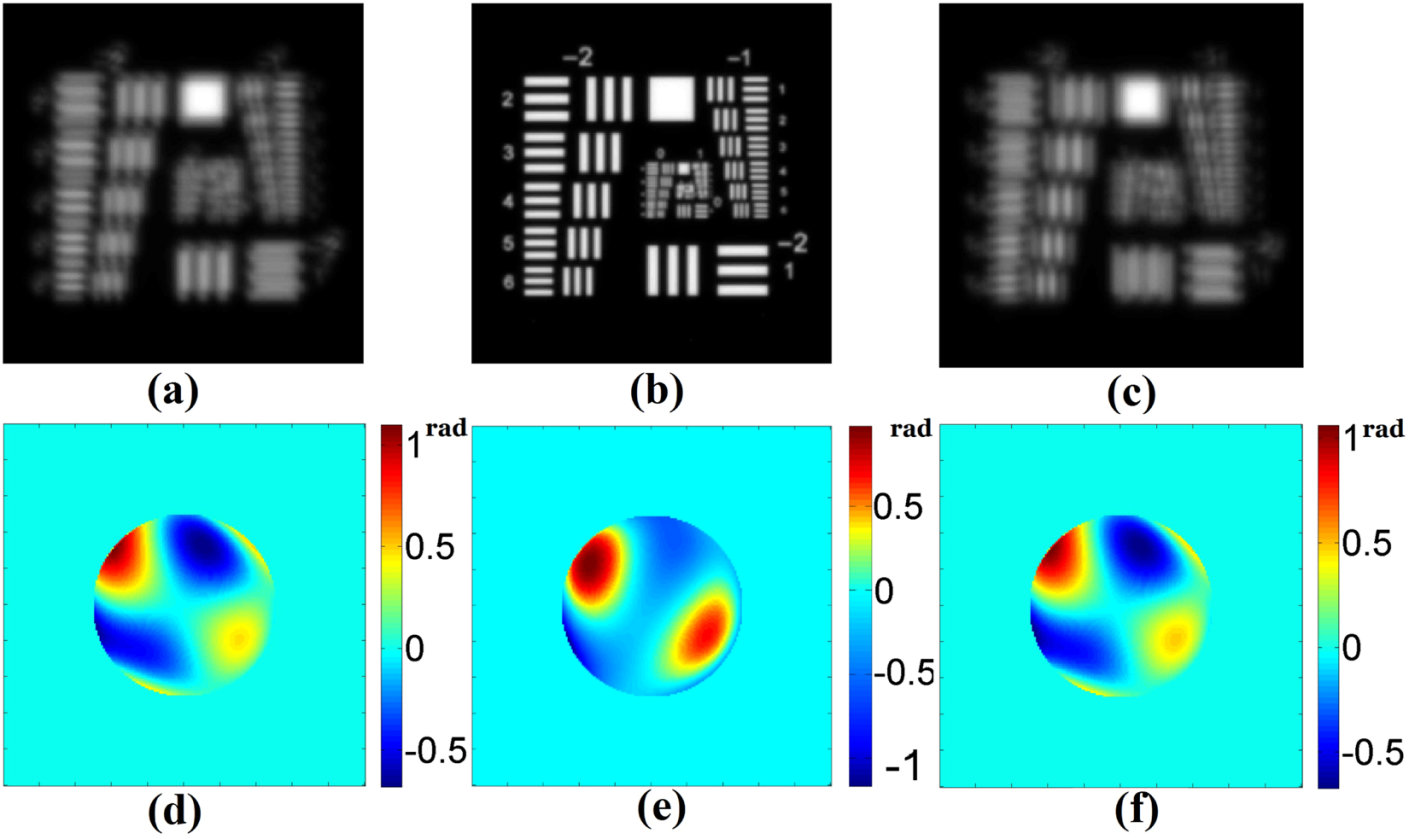
Simulation results by PD-0.5 and PD-BD-0.5 for the aberration listed in Table 1. (**a**),(**b**) and (**c**) are the minus focused image, the focal image and the plus focused image,respectively. (**d**) is the measured phase, (**e**) and (**f**) are the phases reconstructed by PD-0.5 and PD-BD-0.5,respectively.

Moreover, we consider a randomly generated aberration listed in Table 1. We show the CRLBs plotting of PD-0.5, PD-1.0, PD-BD-0.5, PD-BD-0.75 and PD-BD-1.0 in Fig. 3. PD-BDs provides smaller CRLBs than PDs for all Zernike parameters, which indicates stronger capacity of phase retrieval. It is reasonably expected that PD-BD posssesses a higher accuracy than PD. Corresponding simulation results for that aberration by PD and PD-BD are also shown in Table 1 and Fig. 4. The reconstruction accuracy of PD-0.5 is a little better than PD-1.0. Nevertheless, It can be seen that the reconstructed parameters by PD-BDs is apparently more accurate than that by PD-0.5. PD-BD-0.5, PD-BD-0.75 and PD-BD-1.0 provide similar high reconstruction accuracy. RMS errors obtained by PD-0.5 and PD-BD-0.5 are 0.0178*λ* and 0.0032*λ*, respectively. It is also displayed in Fig. 4 that the reconstructed phase by PD-BD-0.5 is obviously more accurate than that by PD-0.5.

**Table 1:** Values(radians) of the coefficients used for CRLB analysis and numerical simulations.

coe	Set	Reconstructed				
		PD-0.5	PD-1.0	PD-BD-0.5	PD-BD-0.75	PD-BD-1.0
*a*_4_	0.1024	0.0299	−0.0416	0.0935	0.0936	0.0947
*a*_5_	0.5409	0.6126	0.6801	0.5410	0.5437	0.5386
*a*_6_	0.2027	0.2413	0.2778	0.2056	0.2058	0.2041
*a*_7_	−0.1453	−0.1857	−0.2083	−0.1686	−0.1535	−0.1501
*a*_8_	−0.0053	0.0195	−0.0146	−0.0216	−0.0117	−0.0104
*a*_9_	−0.3886	−0.3858	−0.3847	−0.3717	−0.3920	−0.3917
*a*_10_	0.1536	0.1674	0.1670	0.1704	0.1631	0.1617
*a*_11_	0.0801	−0.1329	−0.2729	0.0485	0.0457	0.0532
*a*_12_	−0.3007	−0.2697	−0.3247	−0.2938	−0.2988	−0.3024
*a*_13_	−0.4227	−0.3354	−0.3456	−0.4238	−0.4184	−0.4242
*a*_14_	−0.2725	−0.2514	−0.1163	−0.2672	−0.2632	−0.2587
*a*_15_	0.1789	0.1818	0.1558	0.1717	0.1691	0.1725
RMS	0.0549*λ*	0.0568*λ*	0.0626*λ*	0.0545*λ*	0.0547*λ*	0.0546*λ*
RMS error		0.0178*λ*	0.0309*λ*	0.0032*λ*	0.0027*λ*	0.0022*λ*

To further testify the accuracy of PD-BD, we provide the statistical results based on 100 numerical cases. The measured 100 aberrations are randomly generated by setting *a*_*k*_∈[−1, 1], *k* = 4, …, 15 (radians). We define $\overline{CRLB(i)}$ and *RMSE*(*i*) as the average CRLB and the RMS error for the *i* th numerical case, respectively. And the average CRLB and the average RMS error for the 100 numerical cases are defined as $\overline{CRLB}$ and $\overline{RMSE}$, respectively.

5 $$\overline{CRLB(i)}=\frac{1}{12}\sum _{k=4}^{15}CRLB({a}_{k})(i),\,i=\mathrm{1,}\,\cdots ,\,100$$

6 $$\overline{CRLB}=\frac{1}{100}\sum _{i=1}^{100}\overline{CRLB(i)},$$

7 $$\overline{RMSE}=\frac{1}{100}\sum _{i=1}^{100}RMSE(i).$$

We show $\overline{CRLB}$ and $\overline{RMSE}$ obtained by PD and PD-BD in Table 2. $\overline{CRLB}$ obatined by PD-BD-0.75 and PD-BD-1.0 are smaller than that by PDs, which indicates a higher accuracy. Although, $\overline{CRLB}$ obatined by PD-BD-0.5 is a little greater than that by PD-1.0, $\overline{RMSE}$ obtained by PD-BD-0.5 is 0.0143*λ*, nearly two times smaller than that by PD-1.0. Overall, for the 100 numerical cases, PD-BD gains a higher accuracy than PD in 85% of all cases.

**Table 2:** $\overline{CRLB}$ (radians) and $\overline{RMSE}$(*λ*) obtained by PD and PD-BD based on 100 numerical cases.

	Computed				
	PD-0.5	PD-1.0	PD-BD-0.5	PD-BD-0.75	PD-BD-1.0
$\overline{CRLB}$	2.4303E-5	2.3715E-5	2.4076E-5	2.3637E-5	2.3167E-5
$\overline{RMSE}$	0.0186*λ*	0.0306*λ*	0.0143*λ*	0.0148*λ*	0.0150*λ*

With the increasing of the scales of the measured wavefront aberrations, the accuracy of PD and PD-BD will drop gradually. Here, we will compare the performance of PD and PD-BD for wavefront aberrations of the same spatial frequency distribution, but with different scales. The coefficient vector listed in Table 1 is denoted as **coe**. The measured wavefront aberrations of different scales are generated from **coe**, of which each coefficient is multiplied by a same integer. Table 3 lists the RMS errors achieved by PD and PD-BD for wavefront aberrations of different scales. In general, it is can be seen that the accuracy of PD-BD is higher than PD for wavefront aberrations of different scales. It also seems that PD-BD with different amounts of defocus keep satisfying accuracy for wavefront aberrations of different scales.

**Table 3:** RMS errors(*λ*) achieved by PD and PD-BD for wavefront aberrations of different scales.

RMS (*λ*)	RMS errors				
	PD-0.5	PD-1.0	PD-BD-0.5	PD-BD-0.75	PD-BD-1.0
**coe** (0.0549)	0.0178 *λ*	0.0309 *λ*	0.0032 *λ*	0.0027 *λ*	0.0022 *λ*
2 × **coe** (0.1098)	0.0222 *λ*	0.0302 *λ*	0.0067 *λ*	0.0069 *λ*	0.0054 *λ*
3 × **coe** (0.1647)	0.0303 *λ*	0.0521 *λ*	0.0137 *λ*	0.0129 *λ*	0.0101 *λ*
4 × **coe** (0.2196)	0.0375 *λ*	0.0615 *λ*	0.0194 *λ*	0.0207 *λ*	0.0176 *λ*
5 × **coe** (0.2745)	0.0543 *λ*	0.0666 *λ*	0.0301 *λ*	0.0261 *λ*	0.0320 *λ*
6 × **coe** (0.3294)	0.0761 *λ*	0.0820 *λ*	0.0772 *λ*	0.0401 *λ*	0.0508 *λ*

**Figure 5:**
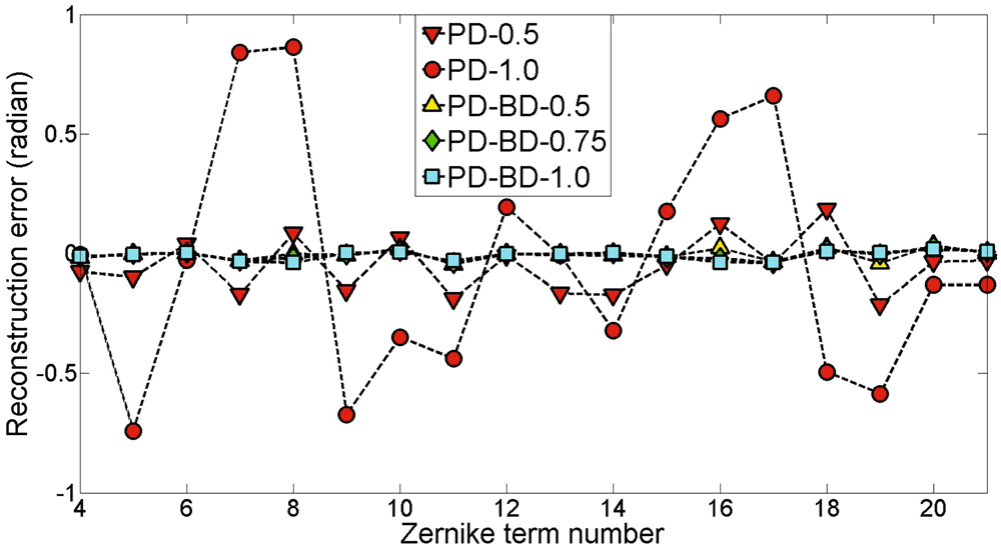
Reconstruction error of each Zerinike coefficient by PD and PD-BD for the set coefficient vector {*a*_4_, …, *a*_21_}.

**Figure 6:**
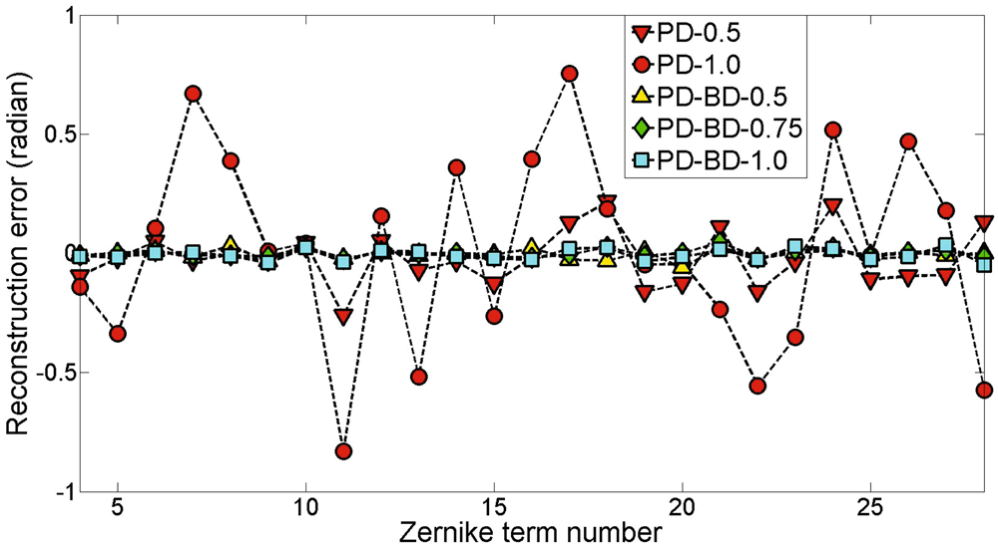
Reconstruction error of each Zerinike coefficient by PD and PD-BD for the set coefficient vector {*a*_4_, …, *a*_28_}.

At last, we will compare the performance of PD and PD-BD for high-frequency wavefront sensing. The measured high-frequency wavefront coefficient vector {*a*_4_, …, *a*_21_} is generated by adding random high-frequency parts to the relative low-frequency wavefront aberration listed in Table 1. And the coefficient vector {*a*_4_, …, *a*_28_} is also generated by adding random high-frequency parts to coefficient vector {*a*_4_, …, *a*_21_}. Reconstruction errors of each Zerinike coefficient by PD and PD-BD for coefficient vectors {*a*_4_, …, *a*_21_} and {*a*_4_, …, *a*_28_} are plotted in Fig. 5 and Fig. 6, respectively.

PD-0.5 and PD-1.0 are obviously less accurate than that by PD-BDs. PD-BD-0.5, PD-BD-0.75 and PD-BD-1.0 provide similar high reconstruction accuracy, nearly five times higher than that by PD-0.5. With the addition of high-frequency parts of the measured wavefront aberration, the reconstruction accuracy of PD-0.5 drop gradually. For coefficient vectors {*a*_4_, …, *a*_15_}, {*a*_4_, …, *a*_21_} and {*a*_4_, …, *a*_28_}, RMS errors abtained by PD-0.5 for different high-frequency wavefront aberrations are 0.0178*λ*, 0.0282*λ* and 0.0305*λ*, respectively. By comparison, RMS errors abtained by PD-BD-0.5 for the same wavefront aberrations are 0.0032*λ*, 0.0052*λ* and 0.0054*λ*, respectively. Compared with PD, it seems that the accuracy of wavefront sensing by PD-BD is less sensitive to the number of Zernike polynomials used and the diversity amplitude, which is a notable advantage.

### Experimental results

**Figure 7:**
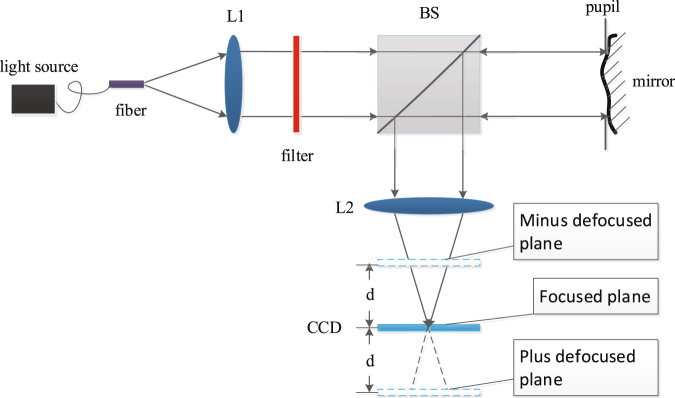
Simplified block diagram of the verification experiment system.

To further examine the accuracy of wavefront sensing with PD and PD-BD, we perform a simple verification experiment. The simplified block diagram of the optical system used here is shown in Fig. 7. The equivalent focal length *F* is 300 *mm*, the pupil diameter *D* is 10 *mm*, and the CCD pixel size is 5.2 *μm*. A common halogen lamp is selected as the light source. A fiber bundle is used as the extended object to be reconstructed. The measured wavefront aberration is randomly generated by a homemade deformed mirror with seven actuators. To obtain quasi-monochromatic images, a filter(632.8 *nm*) is located at the collimated beam. The regularization parameter *γ* is empirically set to be 5.0 × 10^−3^.

We move the CCD by the distance ±*d* to get the plus defocused image and the minus defocused image. The corresponding peak-to-valley optical path difference Δ^26^ is equal to

8 $${\rm{\Delta }}=\frac{d}{8{(F/D)}^{2}}.$$

We choose *d* such that Δ = ±0.5*λ*, so the distance *d* here is 2.279*mm*. The measured object takes the area of 450 × 450 pixels and the exposure time is 20 *ms*. To get rid of the tilts aberrations induced by moving CCD, we match the plus defocused image and the minus defocused image by an image matching algorithm call speeded-up robust features (SURF)^27^. So, similar as our simulations, we ignore piston and tilt aberrations *a*_1_ − *a*_3_. The computed parameter vecter is set as ***a*** = [*a*_4_,*a*_5_, …, *a*_28_].

To examine the accuracy of wavefront sensing with PD and PD-BD, we will compare the detailed clarity of the reconstructed object images. We denote the wavefront coefficients reconstructed by PD and PD-BD as **PD-coe** and **PD-BD-coe**, respectively. With the same input images, or the same formula for object reconstruction, we put **PD-coe** and **PD-BD-coe** into

9 $$O({\bf{u}})=\frac{{H}^{\ast }({\bf{u}})I({\bf{u}})+{H}_{+d}^{\ast }({\bf{u}}){I}_{+d}({\bf{u}})}{|H({\bf{u}}){|}^{2}+|{H}_{+d}({\bf{u}}){|}^{2}+\gamma },$$

respectively. The reconstructed object images are then obtained by taking the inverse Fourier transform of *O*(**u**). Thus, the only difference for the two reconstructed object images are the wavefront coefficients reconstructed by PD and PD-BD.

**Figure 8:**
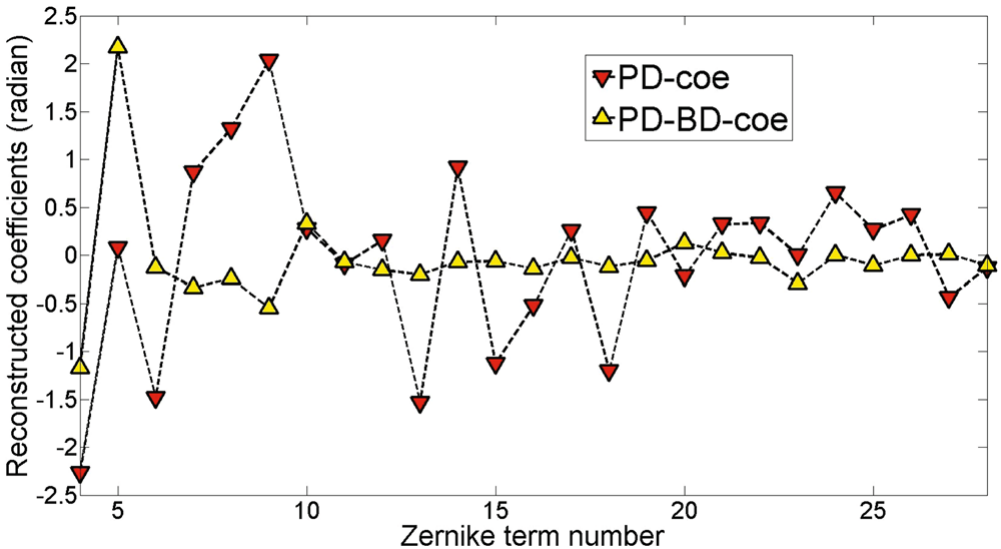
Reconstructed wavefront coefficients by PD and PD-BD for the verification experiment.

**Figure 9:**
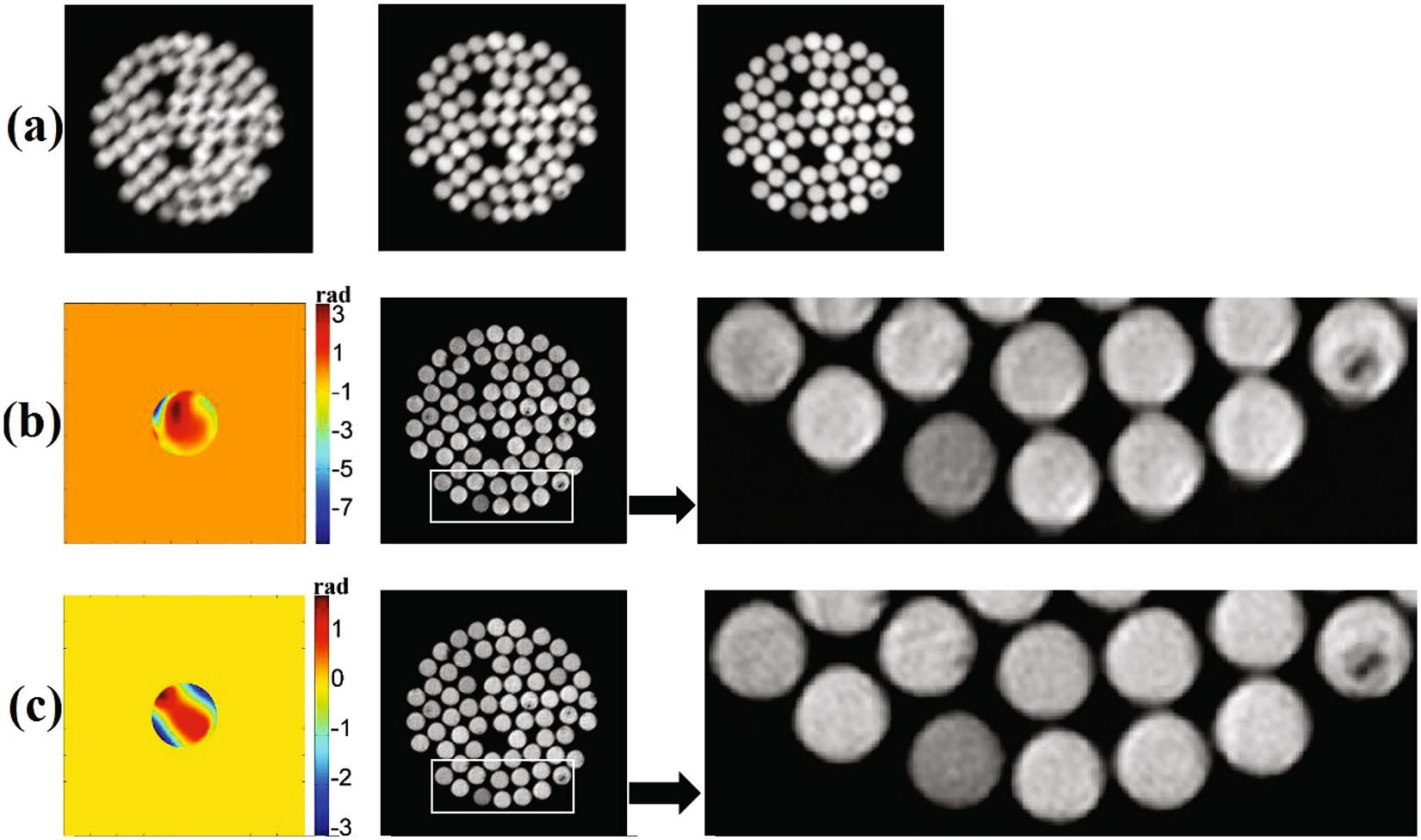
(**a**) contains the minus defocused image, the focused image and the plus defocused image. (**b**) and (**c**) contain reconstructed wavefront and object image by **PD-coe** and **PD-BD-coe**, respectively.

It can been seen in Fig. 8 that **PD-coe** and **PD-BD-coe** are quite different, especially for Zernike coefficients *a*_5_ − *a*_15_. The big difference probably means one of them is much more precise than the other one for wavefront sensing. We show from left to right the minus defocused image, the focused image and the plus defocused image in Fig. 9(a). Figure 9(b) shows the wavefront aberration reconstructed by PD and object image reconstructed by Eq. (9). Figure 9(c) shows the wavefront aberration reconstructed by PD-BD and the object image reconstructed by Eq. (9). From the partial enlarged view of Fig. 9(b), apparent distortions for each single fiber can be found, which means there are still non-negligible and uncorrected system aberrations. While the object image reconstructed by **PD-BD-coe** possesses greater clarity than that by **PD-coe** for each single fiber. We think our experiment results have proved indirectly that PD-BD possesses higher accuracy of wavefront sensing than PD.

## Discussion

For the example of a astigmatism system aberration *a*_5_ = 1.5 radian, PD-BD provides a steeper gradient near the minimum extreme point than PD. A steep gradient of PD-BD near the minimum extreme point can lessen calculative burden and improve the accuracy of wavefront sensing. For the simulations of wavefront sensing with increasing scales of wavefront aberrations, the accuracy of PD and PD-BD drop gradually. Taken together, for different scales of wavefront aberrations, the accuracy of PD-BD is mainly higher than that of PD. With the addition of high-frequency parts of the measured wavefront aberration, the reconstruction accuracy of PD drop gradually. PD-BD could maintain essential reconstruction accuracy for high-frequency wavefront sensing. Compared with PD, it seems that the accuracy of wavefront sensing by PD-BD is less sensitive to the number of Zernike polynomials used and the diversity amplitude, which is a notable advantage for engineering applications. For the simple verification experiment, we have affirmed the fact that PD-BD possesses a higher accuracy of wavefront sensing than PD.

We have statistically compared the CRLB of PD and PD-BD, which indicates PD-BD has a stronger capacity of phase retrieval than PD. In our opinion, the in-depth reason that PD-BD possesses a higher accuracy than PD is due to the shape of the error metric which results in better convergence in the optimization process. For actual engineering application, PD-BD employs two symmetric images of close SNR near the focal plane. This characteristic of PD-BD is favourable to balance necessary amplitude of defocus diversity and necessary SNR of the defocused image. In other words, PD-BD could better ensure the maximum likelihood estimation, which is the foundation of phase diversity technique.

## Methods

In this section, compared with common PD, we briefly describe the bisymmetric defocuses phase diversity. It is supposed that the object is illuminated with non-coherent quasi-monochromatic light, and the imaging system is a linear shift-invariant system^28^. The intensity distribution of the image plane with Gaussian noise can be modeled by the following equation:

10 $$i({\bf{r}})=o({\bf{r}})\otimes h({\bf{r}})+n({\bf{r}}),$$

where ⊗ stands for the convolution operation, **r** is a two-dimensional position vector in the image plane, *o*(**r**) is the object to be found, *h*(**r**) is the point-spread function (PSF) of the optical system, and *n*(**r**) is Gaussian noise. The point-spread function associated with the focused image is given by

11 $$h({\bf{r}})=|{ {\mathcal F} }^{-1}\{P({\bf{v}})\exp \,[j\varphi ({\bf{v}})]\}{|}^{2}.$$

where **v** is a two-dimensional position vector in the pupil plane, *P*(**v**) is the binary aperture function with values of 1 inside the pupil and 0 outside, and *ϕ*(**v**) is the unknown wavefront aberration. ${\mathcal F}$ and ${ {\mathcal F} }^{-1}$ denote the Fourier transform and the inverse Fourier transform, respectively. Similarly, the plus and minus defocused images are given by

12 $${i}_{\pm d}({\bf{r}})=o({\bf{r}})\otimes {h}_{\pm d}({\bf{r}})+{n}_{d}({\bf{r}}),$$

where

13 $${h}_{\pm d}({\bf{r}})=|{ {\mathcal F} }^{-1}\{P({\bf{v}})\exp [j(\varphi ({\bf{v}})\pm {\varphi }_{d}({\bf{v}}))]\}{|}^{2},$$

and the subscript *d* indicates that a known phase diversity ±*ϕ*_*d*_(**v**) has been added to the optical system. The commonly used *ϕ*_*d*_(**v**) is a known defocus aberration. *ϕ*_*d*_(**v**) can be exactly determined by the location of the defocused image plane relative to the focused plane.

**Figure 10:**
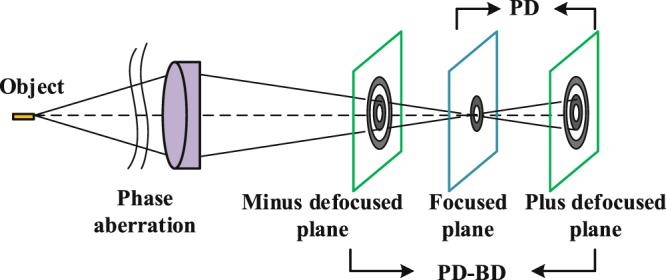
Intensity planes employed by PD and PD-BD.

For common PD, the following error metric.

14 $${E}_{PD}=\frac{1}{2}\sum _{{\bf{u}}}\{|I({\bf{u}})-O({\bf{u}})H({\bf{u}}){|}^{2}+|{I}_{+d}({\bf{u}})-O({\bf{u}}){H}_{+d}({\bf{u}}){|}^{2}\}+\frac{\gamma }{2}\sum _{{\bf{u}}}\{|O({\bf{u}}){|}^{2}\},$$

is usually used to evaluate the mean-squared image intensity difference between the data predicted by optical system and the data actually collected. The second term $\frac{\gamma }{2}{\sum }_{{\bf{u}}}\{|O({\bf{u}}){|}^{2}\}$, called the regularization function, which is usually used to prevent oscillations caused by noise amplification. The parameter *γ* is a small nonnegative constant, which is usually set manually. The major charecteristic of PD-BD is that the measured system aberrtion within the focused plane is reconstructed by two symmetrical defocused planes. In detail, the error metric of PD-BD is

15 $$E=\frac{1}{2}\sum _{{\bf{u}}}\{|{I}_{-d}({\bf{u}})-O({\bf{u}}){H}_{-d}({\bf{u}}){|}^{2}+|{I}_{+d}({\bf{u}})-O({\bf{u}}){H}_{+d}({\bf{u}}){|}^{2}\}+\frac{\gamma }{2}\sum _{{\bf{u}}}\{|O({\bf{u}}){|}^{2}\},$$

where *u* is a two-dimensional spatial frequency coordinate. We show the key difference between PD and PD-BD in Fig. 10. *I*(**u**) is the image intensity distribution of the focal plane in frequency domain. *I*_+ *d*_(**u**) and *I*_− *d*_(**u**) are the image intensity distributions of the plus defocused plane and the minus defocused plane in frequency domain, respectively. *H*(**u**) is the optical transfer function (OTF) of the focused system. *H*_+ *d*_(**u**) and *H*_− *d*_(**u**) are the OTFs of the plus defocused system and the minus defocused system:

16 $$H({\bf{u}})= {\mathcal F} \{|{ {\mathcal F} }^{-1}\{P({\bf{v}})\exp \,[j\varphi ({\bf{v}})]\}{|}^{2}\},$$

17 $${H}_{\pm d}({\bf{u}})= {\mathcal F} \{|{ {\mathcal F} }^{-1}\{P({\bf{v}})\exp \,[j(\varphi ({\bf{v}})\pm {\varphi }_{d}({\bf{v}}{))]\}|}^{2}\},$$

where **v** is the two-dimensional position coordinate in the pupil plane, *P*(**v**) is the binary aperture function with values of 1 inside the pupil and 0 outside. *ϕ*(**v**) is the measured system aberrtion, which is commonly approximated and parameterized by a finite set of Zernike polynomials^29^:

18 $$\varphi ({\bf{v}})=\sum _{j=4}^{K}{a}_{j}{Z}_{j}({\bf{v}}),$$

where *K* is the maximum number of Zernike polynomials. The coefficients *a*_1_ − *a*_3_ stand for piston and tilt of the wavefront aberration, which have no effect on the resolution of image. *ϕ*_*d*_(**v**) is a known defocus diversity phase. Similar to traditional PD, the error metric of PD-BD is minimized by choice of *O*(**u**) when

19 $$O({\bf{u}})=\frac{{H}_{-d}^{\ast }({\bf{u}}){I}_{-d}({\bf{u}})+{H}_{+d}^{\ast }({\bf{u}}){I}_{+d}({\bf{u}})}{|{H}_{-d}({\bf{u}}{)|}^{2}+|{H}_{+d}({\bf{u}}{)|}^{2}+\gamma },$$

where the subscript * represents conjugate operator. Substitution of *O*(**u**) into *E* yields

20 $$E=\sum _{{\bf{u}}}\frac{|{I}_{-d}({\bf{u}}){H}_{+d}({\bf{u}})-{I}_{+d}({\bf{u}}){H}_{-d}({\bf{u}}{)|}^{2}}{|{H}_{-d}({\bf{u}}{)|}^{2}+|{H}_{+d}({\bf{u}}{)|}^{2}+\gamma }\mathrm{.}$$

The error metric *E* is therefore independence of any object estimate. For a given Zernike parameters **a** = [*a*_4_,*a*_5_, …, *a*_*K*_], the error metric *E*(**a**) can be calculated. Optimization process of PD-BD is to find the optimal parameter set *a* for which the error metric is globally minimum. The measured system aberration is then reconstructed by Eq. (18). And the reconstructed object image is obtained by taking the inverse Fourier transform of *O*(**u**).
